# Understanding complexity – the palliative care situation as a complex adaptive system

**DOI:** 10.1186/s12913-019-3961-0

**Published:** 2019-03-12

**Authors:** Farina Hodiamont, Saskia Jünger, Reiner Leidl, Bernd Oliver Maier, Eva Schildmann, Claudia Bausewein

**Affiliations:** 1 Department of Palliative Medicine, Munich University Hospital, LMU Munich, Marchioninistr. 15, 81377 Munich, Germany; 2 Research Unit Ethics, University Hospital Cologne, Cologne, Germany; 3 Cologne Center for Ethics, Rights, Economics, and Social Sciences of Health, University of Cologne, Cologne, Germany; 4 Helmholtz Zentrum München, German Research Center for Environmental Health, Institute of Health Economics and Health Care Management, Munich, Germany; 5 Ludwig-Maximilians-Universitaet Munich, Munich School of Management, Institute of Health Economics and Health Care Management & Munich Centre of Health Sciences, Munich, Germany; 6 St. Josephs-Hospital, Department of Palliative Medicine and Interdisciplinary Oncology, Wiesbaden, Germany

**Keywords:** Palliative care, Complexity, Complex adaptive systems, System theory, classification, qualitative research

## Abstract

**Background:**

The concept of complexity is used in palliative care (PC) to describe the nature of patients’ situations and the extent of resulting needs and care demands. However, the term or concept is not clearly defined and operationalised with respect to its particular application in PC. As a complex problem, a care situation in PC is characterized by reciprocal, nonlinear relations and uncertainties. Dealing with complex problems necessitates problem-solving methods tailored to specific situations. The theory of complex adaptive systems (CAS) provides a framework for locating problems and solutions.

This study aims to describe criteria contributing to complexity of PC situations from the professionals’ view and to develop a conceptual framework to improve understanding of the concept of “complexity” and related elements of a PC situation by locating the complex problem “PC situation” in a CAS.

**Methods:**

Qualitative interview study with 42 semi-structured expert (clinical/economical/political) interviews. Data was analysed using the framework method. The thematic framework was developed inductively. Categories were reviewed, subsumed and connected considering CAS theory.

**Results:**

The CAS of a PC situation consists of three subsystems: patient, social system, and team. Agents in the "system patient" are allocated to further subsystems on patient level: physical, psycho-spiritual, and socio-cultural. The "social system" and the "system team" are composed of social agents, who affect the CAS as carriers of characteristics, roles, and relationships. Environmental factors interact with the care situation from outside the system. Agents within subsystems and subsystems themselves interact on all hierarchical system levels and shape the system behaviour of a PC situation.

**Conclusions:**

This paper provides a conceptual framework and comprehensive understanding of complexity in PC. The systemic view can help to understand and shape situations and dynamics of individual care situations; on higher hierarchical level, it can support an understanding and framework for the development of care structures and concepts. The framework provides a foundation for the development of a model to differentiate PC situations by complexity of patients and care needs. To enable an operationalisation and classification of complexity, relevant outcome measures mirroring the identified system elements should be identified and implemented in clinical practice.

**Electronic supplementary material:**

The online version of this article (10.1186/s12913-019-3961-0) contains supplementary material, which is available to authorized users.

## Background

### Palliative care and the need for differentiation

The World Health Organisation (WHO) defines palliative care as an ‘approach that improves the quality of life of patients and their families facing the problem associated with life-threatening illness, through the prevention and relief of suffering by means of early identification and impeccable assessment and treatment of pain and other problems, physical, psychosocial and spiritual’ [1]. Internationally, there is no uniform way to describe different levels of palliative care but a frequently used approach is to distinguish between generalist and specialist palliative care [2]. Generalist palliative care is provided by primary carers in the community and the hospital. The more resource intensive specialist palliative care aims to support persons with complex care needs and is provided by specially trained professionals in multidisciplinary teams [2, 3]. Patients’ needs can be diverse and vary from symptom relief to information needs and autonomy to make decisions, to psychosocial support for coping with their disease, or spiritual and existential questions. The patients’ relatives, often also called “carers”, are in the dual position of providing care to the patient and being recipients of support [4]. Carers’ needs are often high with respect to their psychological burden, practical support including care instructions, general information and information on pain management [4].

Facing demographic change and an annual increase in deaths, a substantial growth in demand for specialised palliative care is expected [5]. Resources are limited in every health care system. Demographic change and increase in old and comorbid patients will challenge health care systems [6] and especially palliative care [5]. To meet economic challenges and enable a just and efficient allocation of resources, palliative care – including the funding systems for palliative care – should be based on patients’ needs rather than e.g. only diagnoses or prognoses, as currently in most countries [7]. Therefore, approaches are necessary to differentiate patients in need of more resource-intensive specialist palliative care from those for whom a more generalist approach is sufficient. To grade the nature of a patient’s situation and the extent of the resulting care demands, patients, symptoms, care situations, family needs, and other factors are often described or defined by the concept of complexity. In Australia, complexity of palliative care needs has been shown to mirror both resource use and costs [8, 9]. The Australian data show that resource use is best predicted by the factors “phase of illness”, “functional status”, “problem severity”, and “age”. Based on these findings, the Australian National Sub-acute and Non-acute Patient (AN-SNAP) classification was developed and meanwhile integrated in the funding system for palliative care [10]. Other approaches to grade palliative care patients according to their complexity were recently developed in Spain [11, 12]. These approaches provide a promising foundation for theoretical modelling and clinical application which is necessary as there is no common understanding of the definition and operationalization of complexity in palliative care yet.

### The complex system

Glouberman and Zimmerman described three different types of problems: simple (e.g. a recipe), complicated (e.g. sending a rocket to the moon) and complex (e.g. raising a child) [13]. Complex problems like raising a child can contain simple and complicated problems, but they cannot be reduced to those. A crucial criterion of complex problems is the non-linearity of their relations. Also, complex problems are not static – they change over time with changing conditions. Accordingly, complex problems come along with ambiguity and uncertainty. In complex problems formulas and rules can contribute only little to the solution of the problem [13]. The interdependences, non-linearity of cause and effect, and the dynamics of complex problems entail that each complex problem, like every child or every patient and by that every care situation, is unique and requires a different knowledge. Prior experiences with similar problems provide a framework to interpret current problems. However, experience does not guarantee that behaviour leads to the desired outcome. Dealing with complex problems needs a certain method of problem solving [14]. This again requires a fundamental knowledge of the complex problem at present.

System thinking provides a framework in which complex problems and their solutions can be located, and supports an in-depth understanding of the complex problem. A comprehensive theoretical approach is the theory of Complex Adaptive Systems (CAS). CAS theory is not grounded in a specific discipline but is used in a variety of thematic areas [15].

In the context of health care, Plsek and Greenhalgh describe a CAS as *‘a collection of individual agents with freedom to act in ways that are not always totally predictable, and whose actions are interconnected so that one agent’s actions change the context for other agents.’* ([13], p., 625). Apart from the variety of interacting agents, characteristics of a CAS are the concepts of adaptation, emergence, and self-organisation, as well as the concept of attractors and contextuality. A short description of these characteristics follows.

#### Agents

A CAS consists of a variety of elements, called ‘agents’. In their actions, agents follow sets of internal rules or schemes [16]. These serve the agents as reference points for their behaviour and can be applied to new situations instead of assessing each possible situation with an individual rule [17].

#### Interactions

Complex systems cannot be reduced to the sum of agents forming the system. The focus is rather on the interactions between the agents since these are causal for the system’s behaviour. Because of the variety of relationships and their non-linear character, the system behaviour is generally not predictable.

#### Emergence

A decisive characteristic of CAS is emergence. New behaviour and interactions emerge on the level of single agents and the overall system. Also, agents can be eliminated or new agents emerge as a consequence of interactions.

#### Adaptation

Closely linked to the concept of emergence is the system’s ability to adapt. CAS and their agents react to the environment, are able to learn and adapt their behaviour to new circumstances [17].

#### Self-organisation

Since all agents’ interactions influence the system behaviour, a centralized control of the system is ruled out. Not one agent controls the system behaviour – control is decentralized in terms of self-organisation.

#### Attractors

The system can adopt a limited number of states. These successive states which the system adopts over the course of time are called attractors [18].

#### Contextuality

CAS need to be seen in context of their environment. They are part of a super-ordinated system in which they are related to other systems. Accordingly, they themselves consist of subsystems, which again are related with each other. Also, the system behaviour is influenced by signals from outside the system and in turn influences its environment [19].

Social systems are the most complex systems [20]. In the social world, developments always result from a variety of causes which are related and reinforce or override each other [18]. A care situation such as in palliative care comprises humans interacting with each other – the patient, family members, team members and other care providers – and is accordingly a social system composed of social agents. Following system theory in which each system consists of yet other systems of a lower hierarchical order, those can again be groups of persons or, on an even lower hierarchical level, the persons themselves. The understanding of a person as a complex biological system is well established [21] – in a holistic approach it should however be considered that there is more to a person than the biological side. CAS theory offers the opportunity to acknowledge the dynamics and different hierarchical levels of a palliative care situation and may thereby enable a comprehensive understanding of this complex problem.

In the health care context, CAS theory is already well established. The WHO applies CAS theory to health care systems and developed a framework aiming for an understanding of dynamics which shall support change [22]. CAS are suggested for the understanding of health care organisations [16], the success of complex health care interventions [23], and the complexity of clinical consultation situations [24]. Most applications of CAS in health care refer to social systems since they describe interactions between individuals. The theory was also discussed for 'reframing chronic pain as a system opposed to a singularly biological event' and by that proposing a symptom as a CAS [21]. Even though CAS theory is intensively discussed to be an adequate approach to understand complex issues in health care, only little research has been realized in CAS and health care [25]. Regarding palliative care, Munday stresses that the patient can be seen as a system consisting of the common palliative care domains: physical, psychological, social and spiritual [26].

In summary, the term complexity is used to describe issues, situations, persons, and care provided in palliative care. However, the term or concept itself is not clearly defined.

The aims of this study are therefore 1) to describe criteria contributing to complexity of palliative care situations from the professionals’ view and 2) to develop a conceptual framework to gain an understanding of the concept “complexity”, and to identify the elements of a palliative care situation by locating it as a complex problem in a CAS.

## Methods

### Study design

Qualitative interview study using semi-structured expert interviews. The checklist from the COREQ framework [27] was applied to guarantee compliance with high scientific standards. Details are provided in the appendix (Additional file 1).

### Sample and data collection

The sample included both clinical experts and those with an expertise in health policy and financial matters, such as representatives of hospital financial controlling departments, the German Hospital Association, health insurances providers, the German Association for Palliative Medicine, and the German Hospice and Palliative Care Association as well as researchers with a focus on healthcare systems research. Inclusion criteria for participants with clinical expertise were a) a minimum of 5 years working experience in palliative care and b) a management/supervising/leadership role in the service. Inclusion criteria for participants with expertise regarding financial and health policy issues were 1) palliative care as an area of responsibility in the expert’s professional daily routine and 2) a minimum of 2 years working experience in the respective area of responsibility. Purposive sampling was used to ascertain variations of the sample regarding the following criteria: profession, care settings, rural or urban area, university affiliation, and geographical region. The chosen experts allowed to cover the topics complexity, resource needs, and costs in palliative care in Germany from various angles, and hereby to prevent bias due to a one-sided perspective. Most experts were selected based on suggestions from the research team and collaborating partners. Additionally, representatives from the German Association for Palliative Medicine were asked for suggestions.

Interviews were conducted face-to-face with one exception of a telephone interview. The setting was chosen by the respondents and was predominantly his or her working environment.

### Interview guide

Two interview guides were developed for this study – one for clinical experts and one for experts with a health policy and financial background (for English translation of the interview guide see Additional file 2). Apart from complexity, the interview guide also included questions on resource needs and funding of palliative care in Germany. Clinical experts started with the questions on complexity while experts with health policy and financial background started the interview with questions on funding of palliative care followed by the questions on complexity. In each case, the complexity questions started with a general question on complexity of the patient situation. Subsequently, two case vignettes with different levels of complexity were presented in order to encourage further conversation on possible complexity factors. The case vignettes were taken and translated from a project on complexity in palliative care at King’s College London, UK [28].

Both interview guides were developed and discussed within the project team, including health economists, sociologists and widely experienced palliative care professionals. The development of the interview guides followed the four-step procedure offered by Helfferich: collecting, reviewing, sorting, subsuming [29]. The topic guide was discussed in a multidisciplinary research group focusing on clarity of questions and structure. Test interviews were conducted to obtain information on interview duration as well as the working of questions and thematic structure.

### Data management and analysis

All interviews were audio recorded and transcribed verbatim. Transcripts and audio files were encrypted in order to avoid identification of the interview partners. NVivo 10 was used for data management [30]. Only the interview passages referring to complexity of care situations were subject to this analysis. Data was analysed by qualitative content analysis applying the framework method developed by Ritchie and Lewis [31]. The framework was developed inductively in close collaboration of FH, ES, and CB. Coding consistency was ensured by applying a coding guide and the verification of intra-coder reliability (FH) for three, and inter-coder reliability (ES, FH) for five interview transcripts. Responses before and after presentation of vignettes were compared to preclude characteristics to be included in the analysis which were suggested by the vignettes. In the sense of inductive theorizing, the systems approach to the research question became apparent during the interviews and in the first steps of framework analysis (familiarizing and describing). In the process of inductive theorizing, categories and codes of the framework were structured in factors referring to the patient, the social system, the team, and structure. CAS theory was identified to match the ideas from the process of inductive theorizing and was deductively applied to the Framework. Codes were reviewed, subsumed and connected considering CAS theory.

## Results

Overall, 42 interviews, 27 with clinical experts and 15 individuals from an economic/political background, were conducted in the time from June 2015 – October 2015. Interview duration ranged between 19 to 113 min with a mean duration of 58 min. 43/48 invited experts (90%) accepted the invitation. One interview was cancelled on short notice because of a clinical emergency. Reasons for declining were lack of time in four cases and too little experience in palliative care in one case. The distribution of characteristics of the sample is presented in Table 1.

**Table 1:** Participant characteristics

	Clinical experts				Experts with financial and politicy focus			
Participant characteristics (*n* = 42)	total	Physician	Nurse	Social Worker	total	Financial focus	Political focus	Healthcare systems researchers
	*n* = 27	*n* = 16	*n* = 10	*n* = 1	*n* = 15	*n* = 8	*n* = 5	*n* = 2
*Gender*								
Male	13	7	6		11	6	3	2
Female	14	9	4	1	4	2	2	
*Actual work setting (multiple count)*								
Palliative care hospital unit	11	7	4		10	6	4	
General care hospital unit	1	1	0		3		3	
Hospital support team	6	5	1		7	4	3	
Specialized palliative home care	10	5	4	1	7	3	4	
General palliative home care	5	3	2		4		4	
Hospice	4	2	2		4	1	3	
*Experience (*median, range)								
Years of palliative care experience	15, 3–30	20, 10–30	14, 3–25	15	10.5, 1–14	7.5, 1–30	11, 10–27	10, n/a
*University affiliation*								
Yes	5	3	2			Not applicable		
No	22	13	8	1				
*Area*								
urban	22	12	9	1		Not applicable		
rural	5	4	1					
*Geographical region*								
North	4	3		1				
East	4	2	2					
South	13	9	4		5	5		
West	6	2	4					
national					10	3	5	2

Notes Table 1: Some participants were working in multiple care settings, e.g. a specialized palliative home care physician also working in a hospice. The social worker and one nurse were coordinators working at the interface of general and specialized care. One healthcare systems researcher wasn’t able to make an adequate guess in the working-experience with palliative care – the topic was not taken on at a specific point in time but was part of the overall research on healthcare systems

The initial framework consisted of 105 categories and was reduced to 57 system elements and environmental factors when applying CAS theory to the framework. The presentation of the vignettes did not result in additional categories but to a more frequent and in-depth discussion of themes.

**Fig. 1:**
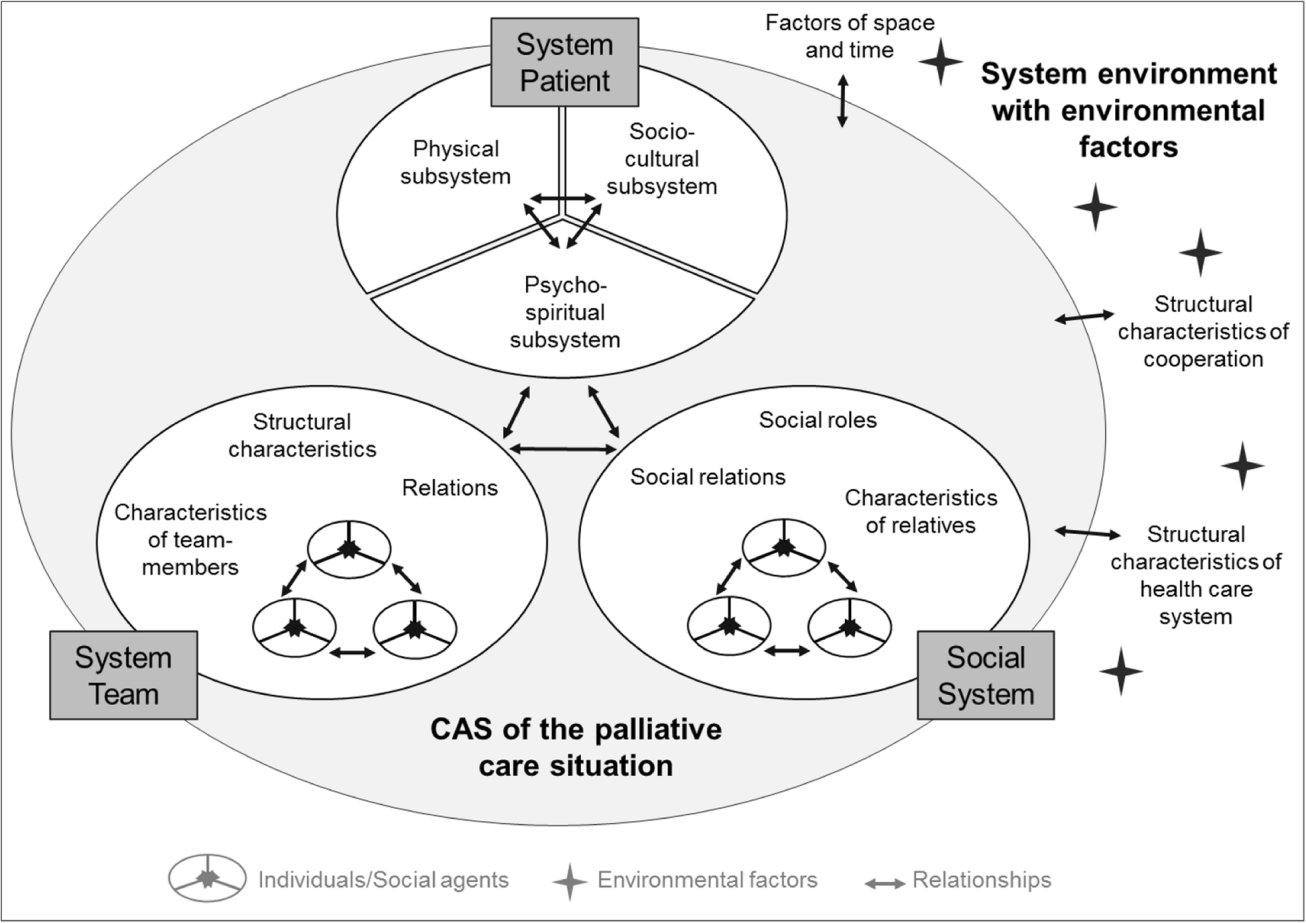
The palliative care situation as a complex adaptive system

Three systems were identified to account for the overall CAS of a palliative care situation: The system patient, the social system, and the system team. System elements from all three systems interrelate with each other as well as with the environment and modulate the overall system behaviour. While the patient system is organised on person level, the social and team system is a collection of social agents (individuals). They affect the CAS of the care situation as carriers of certain characteristics, social roles and relationships (Fig. 1).

Table 2 and Table 3 show an overview of all identified system elements, ordered by (sub)systems and categories, and demonstrate their relations and interactions with each other as well as how these relations are directed. Indirect effects of agents‘ behaviour on other agents are possible even if these do not have a direct mutual relation. Further relations between the simultaneous occurrence of system elements and the system behaviour were described. The simultaneous occurrence of one agent with another one rather influences the system behaviour by co-acting, in the sense that the sole parallel occurrence causes certain behaviours in the system. Co-acting is accordingly also listed in the respective agent descriptions in Tables 2 and 3. Due to space limitations no citations will be used for illustration within the paper. A list with illustrating citations for each system element is provided in the online appendix (Additional file 3).

**Table 2:**
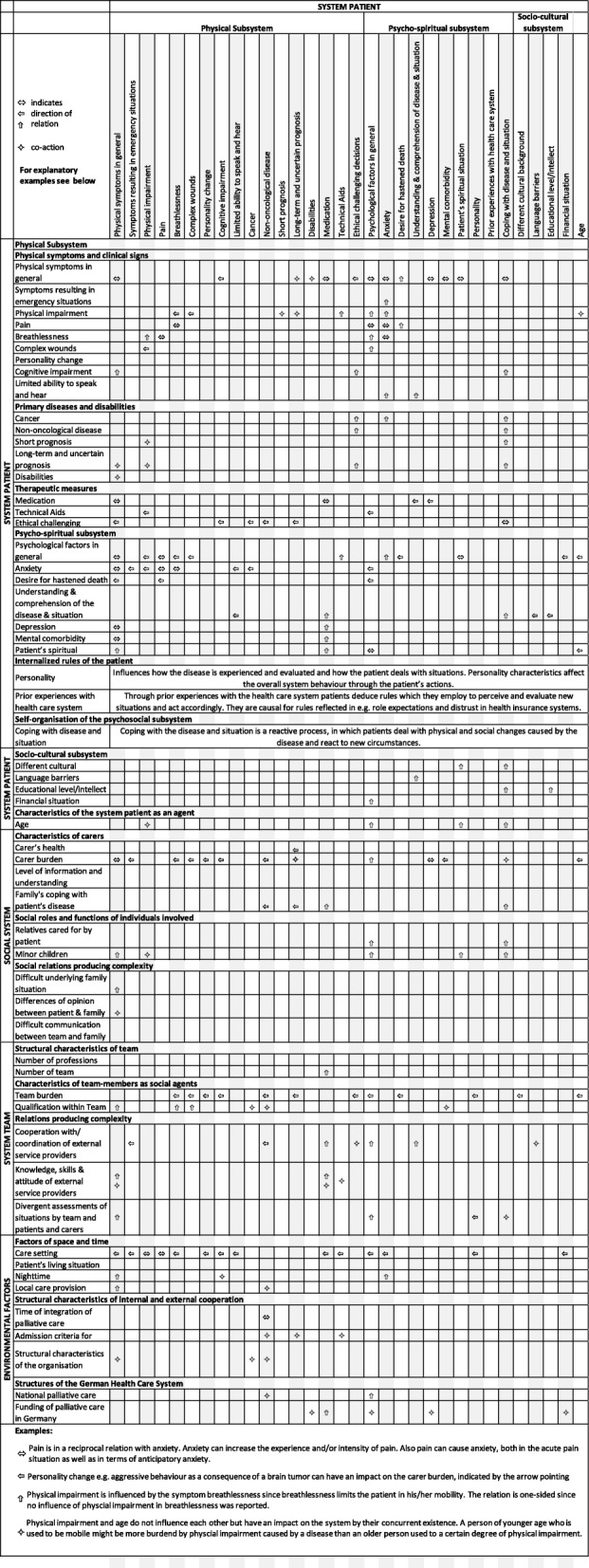
System elements ordered by (sub)systems and categories including their relations and interactions with the subsystem patient

**Table 3:**
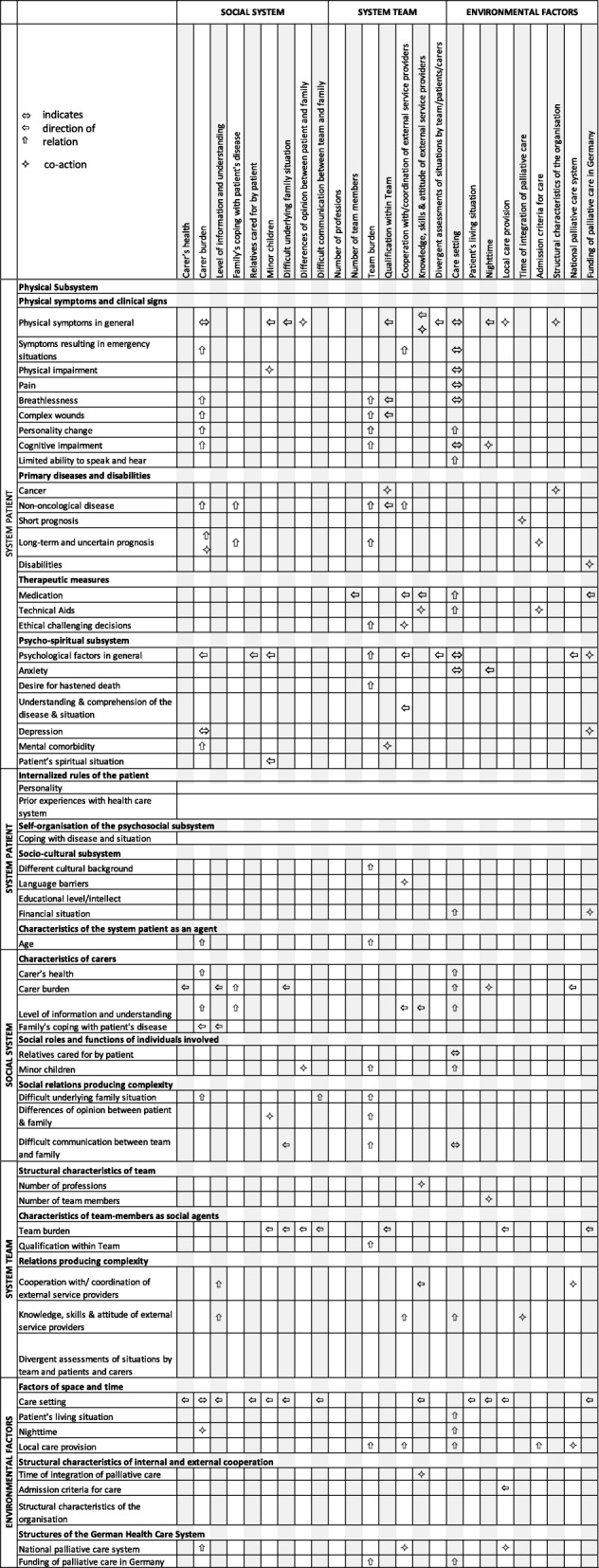
System elements ordered by (sub)systems and categories including their relations and interactions with the “social system”, “system team”, and “environmental factors”

### The patient system

The agents of the patient system were assigned to additional subsystems on patient level: the physical subsystem, the psycho-spiritual subsystem, and the socio-cultural subsystem.

#### The physical subsystem

The physical subsystem includes all agents referring to the patient’s physical dimension. They can be subdivided into three categories: physical symptoms and clinical signs, primary diseases and disabilities, and therapeutic measures. The existence and the effect of agents in the physical subsystem are generally caused by physical, bio-chemical, and technical processes, which can be understood as rules these agents follow.

##### Physical symptoms and clinical signs

Physical symptoms and clinical signs play a major role in the CAS of a palliative care situation. They are the physical manifestation of the progressive disease(s). As agents and due to their relations with other system elements they have decisive impact on the system behaviour, both on a general level and more specifically in terms of increasing complexity of a palliative care situation. On a general level, the patient’s burden of the physical symptoms increases potentially with the number of symptoms. However, a patient can also experience a single symptom as so burdensome that it affects a multitude of other agents and systems and therefore influences the system behaviour of the palliative care situation. For example, breathlessness as a symptom is related to other agents of the physical subsystem (e.g. interdependencies with pain), the psychosocial subsystem (e.g. by causing anxiety which may again increase breathlessness), as well as the social system and team (since breathlessness is experienced to be very burdensome to both). Hence, as an individual symptom, it increases complexity and by that may have limiting effects on home care.

Symptoms and clinical signs which exceeded the ‘symptoms in general’ regarding relations and impact were included in the description of the system as individual agents, e.g. pain, breathlessness, complex wounds, and personality changes.

##### Primary diseases and disabilities

The patient’s primary disease(s) influence the CAS of a palliative care situation through symptoms and the disease trajectory. In non-oncological diseases for example, diagnoses and prognosis are often long and uncertain which may affect the patient’s coping with the disease. In terms of symptoms caused by the disease, diagnosis was considered to be relevant regarding a successful symptom management, e.g. choosing the appropriate medication for a physical symptom which may differ depending on the underlying disease.

##### Therapeutic measures

The category of therapeutic measures includes those agents which intervene with the disease process, such as any medication, technical support or decisions or therapeutic measures. They are the result of a decision which will have consequences on further care and treatment and the course of the disease.

#### The psycho-spiritual subsystem

The psycho-spiritual subsystem involves all system elements referring to the patient’s emotional, spiritual and existential world of experience, e.g. anxiety, desire for hastened death, spiritual situation. Agents follow rules of cognition and emotion, which base on formerly adopted knowledge. In addition to the factors which can be interpreted as agents of the psycho-spiritual subsystem, factors were described which refer to the patient’s personality, such as distinctive personality traits, as well as former experiences with the health care system. These factors can be understood as internalized rules and principles against which patients perceive and assess situations and adjust their behaviour accordingly. Patient’s coping with the disease and situation is also part of the psycho-spiritual subsystem. Coping is a process, which can be understood as adaptive behaviour by which the complex adaptive psycho-spiritual subsystem reacts with self-organization to situations and signals from the environment.

Data indicate strong relations between agents in the psycho-spiritual and physical subsystem. For example, agents, such as anxiety, depression or the patient’s spiritual situation, are affected by physical symptoms such as complex wounds, breathlessness, and pain and vice versa intensify physical symptoms.

#### Socio-cultural subsystem

Factors allocated to the socio-cultural subsystem such as cultural background and language barriers are not actual agents in the classical meaning since they cannot “act”. They are rather characteristics inducing behaviour of the patient as a social agent or have a decisive influence on the behaviour in terms of contextual rules. These characteristics act through the patient as a social agent and influence the system behaviour.

#### Age as a characteristic of the patient as a social agent

In addition to the agents in the three subsystems, the patient’s age was named as a factor potentially influencing the complexity of a palliative care situation. The patient’s age influences interpretation of situations by the patient him- or herself and by other social agents and influences their action; for example, in terms of non-acceptance of a life limiting illness in young age or higher likelihood of identification with a young patient by staff.

### The social system

The social system is composed of several social agents (individuals) who define the system behaviour. The social system influences the overall CAS of the palliative care situation by behaviour and relationships of individual agents as well as by those of the social system as a whole. Social agents´ behaviour follows rules in terms of cognition and emotion as well as social norms. The meaning of the patient’s social system for the CAS of a palliative care situation is at least threefold. First, the social system and its agents are related to agents from the system patient (e.g. the psycho-spiritual subsystem) and influence its behaviour. Second, as part of the unit of care they are also beneficiaries of the care themselves. As such they need to be supported and informed and their varying needs must be addressed. Third, they are simultaneously involved in the patient’s care and are a resource supporting the professional team. Because of the role the social system plays in the patient’s life and the overall CAS of a palliative care situation, its absence also has an impact. The absence of close relatives or friends and the resulting lack of support can affect agents in the other subsystems (patient, team).

Predominantly, the existence of a social system is evaluated positively and beneficial to the palliative care situation. A social system is important for the psycho-spiritual and social support and can provide emotional security. A well-functioning social system being involved in the care of the patient was described to reduce complexity and to relieve the professional team. The social system is only supportive if it is stable. Accordingly, it needs to be supported – resources to maintain the resource social system are required. The existence of a social system also adds to complexity. The more agents act in the social system, the more relationships and behavioural possibilities influence the system behaviour, which influences the degree of complexity of the palliative care situation.

Identified factors were subsumed under the following three categories referring to characteristics of informal carers as social agents and their relationships.

#### Characteristics of carers as social agents

Characteristics of carers as social agents influence the extent of support they can provide in caring for the patient and the amount of care they need themselves. Carers‘needs may even be predominant and require considerably more resources than the patients´ – for instance when family members are overburdened by the situation and/or their own health status.

#### Social roles and functions of individuals involved

Social actors take on various roles and functions, which influence the overall system. Roles described to co-determine the situation’s complexity are defined by the relative’s dependency on the patient, e.g. minor children or older dependents the patient cares for. The responsibility and worries about the care of the dependents after death can be a considerable additional burden to the patient and affect the patient’s psycho-spiritual subsystem, respectively. Also, since dependent relatives cannot take on the patient’s care, they are no practical support for the team. On the contrary, they also need care and support for the planning of this care in the acute illness situation as well as after the patient’s death.

#### Social relations producing complexity

Certain relationships of carers as social agents were described to potentially have major impact on the system behaviour of the palliative care situation. The relationships between carers among each other, between carers and the patient, and carers and the team influence the behaviour of individual social agents, sub-systems and the overall CAS of a palliative care situation. Difficult relations between social agents result in challenging communication between individuals. They increase the need for care resources and may have a burdening effect on the team. For example, conflicts arising from difficult underlying family situations can destabilize the system, and increase the complexity of the care situation and the need of resources.

### The team system

Like the social system, the system team is of a higher hierarchical level than the system patient. It is composed of social agents, the team members, who act upon emotional, cognitive and social rules.

As initiators of therapeutic interventions, the team has direct impact on the system patient. Also, on the social and emotional level, the team reacts to the situation of patients and relatives and is therefore a co-producer of complexity in the care situation. With its behaviour, the professional team reacts to signals coming from the other subsystems: the patients’ and relatives’ needs.

Cooperation within the team results from interactions between many different professionals involved. Due to this variety of actors and their relations, cooperation within the team already implies complexity. Differing opinions between team members regarding patients and their family, as well as uncertainties and ambiguities can be a reason for conflicts within the team. For the team to be able to react with high quality care to the often changing and complex situations, it needs a certain attitude and the ability to react flexibly and communicate promptly. Additionally, for coping with the burden on individual team members and within the whole team, the team itself needs psychosocial support .

The behaviour of the system team and its single team members as care providers has a direct impact on its performance and the quality of care. Described factors can be grouped under the following three categories.

#### Structural characteristics of the team

The system team consists of various social agents providing the care tailored to patients’ needs. Structural characteristics, such as the number of team members, their profession and qualification, affect the team’s performance and accordingly the behaviour of the palliative care situation as a CAS.

#### Characteristics of team members as social agents

Team members are social actors. Their behaviour towards the system patient and the respective subsystems, the social system and agents from outside the CAS of the palliative care situation is influenced by their emotional, cognitive and physical reaction to the situation(s) they are facing and by the abilities and qualification they bring to the situation.

#### Relations producing complexity

Within the relationships between team members and patients and carers, divergent assessment of situations may occur adding to complexity. Also, the team and its individual members are not the only people involved in the patient’s care. Relationships between the team and other professionals as social agents outside the overall CAS of the palliative care situation were described to have influence on the work delivered by the team as well as on the system behaviour of the system patient and social system. For example, experiences and attitudes of external professionals can lead towards differing information communicated to patients and family, which can result in insecurities and burden.

### Environmental factors

Additional to the three systems of the overall CAS of a palliative care situation, factors from outside the actual palliative care situation influence the system behaviour. These factors are generally part of a system of higher hierarchical level and can be subsumed under three groups of factors: Factors of space and time, structural characteristics of internal and external cooperation, and structures of the German health care system. For example, institutionalisation in health care may have implications for complexity, particularly since dynamics of time and decision-making of these systems are highly diverse from actual patient care - such as local capacities of care provision or reimbursement procedures of statutory health insurance companies.

## Discussion

To our knowledge, this is the first study describing a care situation as a CAS and analysing the elements explaining complexity, not only in palliative care but in health care in general. The criteria contributing to complexity of palliative care situations from the professionals´ perspective could be allocated to three systems of the overall CAS of a palliative care situation: the system patient, the social system and the system team as well as to environmental factors. The developed conceptual framework reflects the holistic approach of palliative care and highlights that elements, such as symptoms, persons or certain family relations, cannot be understood independently and separated from the overall system of the palliative care situation.

It could be argued that the results merely mirror the domains of care (physical, psychological, social and spiritual) incorporated by the holistic model of palliative care, and that knowledge of this model might even have limited the participant’s answers to these domains. The findings are certainly shaped by the domain-based understanding of palliative care. They are, however, not limited to those. The experts not only described system elements and their relationships associated with these domains, but also additional aspects of complexity, such as dynamics and interactions of these elements as well as environmental factors and team aspects. The findings suggest that the existing domain-based model of palliative care does not comprehensively describe complexity of a care situation, since it does not incorporate these additional aspects of complexity.

The understanding of the palliative care situation as a CAS supports and supplements findings from other studies on complexity and palliative care. On the patient level, Pask’s et al. findings of applying Bronfenbrenner’s Ecological Systems Theory to the complexity of patients’ and families’ needs also show that there is more to complexity in palliative care than the physical, psychological, spiritual and social dimension [28]. They identified additional components of complexity, such as dynamics, relationships, influence on the societal and organisational level, which agree with the conceptual framework presented in this paper. The results from Tuca et al. indicate that interactions between the variables included in their study predicted complexity better than the sole variables [12]. The Spanish research group suggests complexity to be a multidimensional construct complying with complexity theory. In terms of CAS, Ciemins et al. pointed out that it is supportive for the work of the multiprofessional team to comprehend patients, families, teams and organisations as CAS [32]. CAS has been suggested as an appropriate conceptual framework to understand team processes and support team development [32, 33]. Defining the palliative care situation as a CAS provides a systemic view in which the patient and his or her relatives are still central elements, but in addition, the team assumes a position within the care situation. Besides, it merges various hierarchical levels and enables the understanding of lower hierarchical level agents such as symptoms acting and interacting with elements of higher hierarchical level, such as the team. The application of CAS theory supports a better understanding, building the theoretical foundation upon which to develop a situation sensitive method of problem-solving – not only in the palliative care context. The findings of this study depict the CAS of a specific problem and show how other problems of health care can also be framed by systems thinking.

### Using CAS framework to influence system behaviour

Some of the identified system elements and environmental factors do not refer to the patient but are imposed by the organisation and management of care. Structural and process characteristics on the level of the team, the care organisation or the health care system influence the system behaviour. Acknowledging the effect of structural and process characteristics on the complexity of care situations enables the development of strategies to influence the system behaviour and outcomes by reshaping these characteristics, for example by setting appropriate incentives in payment for care. Changes regarding the timing of integrating palliative care in the care trajectory may for instance have an impact on the continuity of care, enable easier transitions for patients and carers and thus result in an increased quality of care [34, 35]. In consequence, this could potentially decrease the complexity of a care situation. The specification of quality criteria for care facilities on a structural level, such as the number of team members and professions within the team, enables the creation of a constant on the structural level. This would enable the evaluation and comparison of the complexity of a care situation independent of differences on the organisational level.

Pype et al. pointed out that in social systems such as a palliative care team, the agents’ internalized rules are subject to change [36]. Considering the CAS of a palliative care situation, this also applies to other social agents involved: the patient and individuals in the social system. In social agents, the internalized emotional, cognitive and social rules are not static and are subject to change, if e.g. a person reflects on those rules and consciously changes them or if rules are dictated and changed by the environment [36]. For example, legal specifications, documentation requirements, or funding structures provide rules, which the team follows. If external rules change (e.g. a legislative change), the team will adapt its behaviour which will in turn have an impact on the overall CAS of a palliative care situation. While Pype et al. focus on how this understanding may influence team behaviour and can be used for team development purposes, the team’s integration in the overall CAS of a palliative care situation suggests that these changes will also have an impact on other system elements in the realm of the patient and social system and therefore the overall care situation.

Looking at different system elements (and environmental factors) referring to structure may help to discover potential for change and improvement of quality of care.

### Using CAS framework for differentiation of patients’ needs

CAS theory not only offers a comprehensive conceptual framework for problem solving in palliative care. It can also be used to support the development of a systematic approach to differentiate patients according to their need for general and specialized care. The CAS of a palliative care situation provides potential criteria for the classification of complexity. Since the emphasis of CAS is on relations between elements, criteria included in a classification need to account for that. In fact, a classification such as the diagnosis related groups (DRG) system in health care taking only diagnoses and procedures into account is too reductionist to meet the multifaceted nature and relations of the palliative care situation. Therefore, it is unfit to mirror complexity and resource needs. The development of a model or classification of complexity certainly requires the reduction and simplification of information to make it measurable. This holds two major challenges: 1) Not all elements identified to add to complexity are measurable. Elements such as the patient’s personality, prior experience with the health care system or a difficult underlying family situation may have a major impact on the system behaviour but cannot be assessed easily and accordingly cannot be included in the modelling. 2) The large number of elements and relations needs to be reduced to a manageable number for assessment which still describes a situation comprehensively.

The in-depth understanding of interdependences may help to find alternative ways of incorporating system elements which cannot be measured or whose measurement would be too resource-intensive. The knowledge of their influence on other system elements allows involving them indirectly in a classification. Accordingly, the understanding of interdependences can be used to reduce the number of variables without oversimplifying information.

According to complexity science, the degree of complexity depends on the number of system elements, such as symptoms and social agents, environmental factors, and the quality of the relations with each other. Statistical modelling methods need to account for that. Methods arising from the traditional reductionist paradigm of science aiming for principles which follow the assumption of linear relations are not appropriate to deal with complex problems since they strongly reduce and oversimplify information [18, 19, 37, 38]. An appropriate method needs to reflect relationships and build on multivariable analysis methods such as applied in the development of the Australian palliative care classification [8].

Three of the four factors used in the Australian classification – functional status, problem severity and age – are also represented in the elements of the CAS and could be used as a starting point for a German classification. Phase of illness as the factor predicting resource use best in the Australian studies was not directly identified in our data. The concept of “phase of illness” could, however, be understood as a result of the presence of and interactions between the identified elements and factors.

### The use of attractors in modelling a patient classification

With the idea of attractors, CAS theory offers an additional approach to assess complexity of care situations. Attractors are states which the system will adopt over the course of time and through the system behaviour. The system behaviour is the result of interacting agents.

The data in our study did not provide any states which could be interpreted as attractors of the CAS of a palliative care situation. However, attractors are defined by the problem and by the system tailored to the problem. For example, in the psycho-spiritual subsystem, “coping with disease” was acknowledged as the process of the subsystem’s self-organisation. It could be argued that the stages of coping with the disease can be understood as the attractors of the subsystem. On the higher level of the system patient, phase of illness, as proposed by Masso et al., and used in the Australian AN-SNAP classification [10, 39], could be defined as attractor. While the disease progresses, the patient will change between these phases: stable, unstable, deteriorating and dying. Hence, phases of illness are states which will be adopted by the patients, independent from the disease, symptoms, social situation, etc. The phases refer to the patient as well as the carers and reflect the concept of the unit of care inherent to palliative care. The description of the phases includes several references to the carers’ situation and how it may influence the care situation [39]. Furthermore, phases of illness do not follow a predefined order. Patients and care situations can move between phases in any direction [39]. The patient and the respective care situation will always be in one of the phases or in transition between two phases. This complies with the concept of attractors. Hence, the CAS concept of attractors enables the inclusion of a measurable variable reflecting several elements and relations, in this case phase of illness, into the concept and the classification of complexity with respect to patients in need for palliative care.

Since attractors are a construct, it is not possible to determine which agents and relations are covered by them. Phase of illness refers to the patient and the social system. The system team and environmental factors are not considered in the concept. Accordingly, the use of phase of illness as the sole predictor for resource use would not be appropriate since it entails the risk of excluding relevant system elements, environmental factors, and relations.

### Implications for practice, policy and future research

The approach applied in our analysis will contribute to overcoming the present arbitrariness in the use of the term and the concept of complexity, and thereby lay a foundation for future theoretical modelling and clinical applications. In terms of a necessary operationalisation of complexity, a set of relevant outcome measures needs to be identified which can and should be clinically applied. As shown in the Australian AN-SNAP model such outcome measures can be used for a classification to differentiate patients according to their needs, benchmark palliative care services [40], and as a basis for a financing model [10]. Our data suggest that, in accordance with the developments in Australia, these outcome measures should cover problem severity, functional status, and potentially phase of illness. The current version of the AN-SNAP classification consists of 30 classes, 21 of which refer to adult patients [41]. Furthermore, classes are divided by in-patient and home care situations, reflecting the relevance of the care setting as acknowledged by the environmental factor “care setting” in our findings.

In Australia the *Palliative care problem severity* score is used for the classification, measuring pain, other symptoms, psychological and spiritual distress of the patient and carer burden [42]. In Germany, the *Integrated Patient Outcome Scale* (IPOS) and the *Symptom and Problem Checklist of the German Hospice and Palliative Care Evaluation* (HOPE) are validated outcome measures well established in clinical care [43–45]. Especially IPOS can be considered a suitable instrument to routinely measure factors influencing the complexity of a palliative care situation. Apart from questions regarding the distress caused by physical symptoms, IPOS also covers questions regarding the psychological and spiritual situation of the patient as well as practical problems and carer burden [43, 44]. Also, the IPOS offers a more comprehensive problem assessment than the *Palliative care problem severity* score, since it explicitly covers other symptoms, such as breathlessness, which have major effect on complexity. Physical impairment, also included in the AN-SNAP classification, can be measured by the *Australian Karnofsky Performance Status* or the *20-point Modified Barthel Index* [46–48]. These already established outcome measures offer starting points for the measurment of system elements identified in this study, which could be involved in a classification by scores or categories.

The Australian classification can be considered as a successful example for the development and use of a classification and can be an orientation point for the development of a classification in Germany and other countries. However, as systems thinking suggests, even a successfully used classification cannot be seen independently from its superordinate system. The Australian classification cannot simply be transferred to other countries due to differences in health care systems, organisations and work place culture. Further research is needed in Germany and other countries to enable classifications fitting the respective national system characteristics.

Furthermore, our findings address the demand for a stronger theoretical foundation of health services research. Complex problems cannot be represented adequately by a scientific understanding of linear causalities usually prevailing in medical research. Future research concerning complexity in palliative care may benefit from drawing on the theoretical model of CAS throughout all phases of the research process, including the definition of the research question, the identification, operationalisation and measurement of relevant parameters, and the interpretation of findings. The consideration of the CAS as a theoretical framework may be particularly useful in the development of interventions, and in implementation research, since the anticipation and understanding of complex interactions will be vital for the successful realisation of innovation and change in healthcare. This may also involve a stronger focus on healthcare providers such as teams or individual healthcare professionals as agents in the care system, contributing to the outcome of care, and hence constituting a relevant research variable.

### Strengths and limitations

To our knowledge, this is the first study to systematically analyse the definition and operationalisation of complexity in palliative care using the framework of complex adaptive systems.

A particular strength of this study was the relatively large sample including stakeholders with diverse perspectives on palliative care, represented by clinical experts as well as experts with political and economic background. The two sample groups (group a and group b) and the heterogeneity of the experts included regarding the selection criteria (profession, care setting, rural or urban area, geographical region and university affiliation of the centre) were selected to ascertain variation in perspectives and hereby reduce potential bias.

A limitation of this study is that it focused on the professional carers’ perspectives on complexity only. Due to resource limitations patients’ and carers’ perspectives were not included in this analysis and their needs incorporated in the results are based on the professionals’ perspective. Besides, the study was only conducted in one country. However, in the meantime, a study exploring the perspective of patients and carers in the UK has been published. The results confirm our findings and do not show any additional elements not represented in our data [28].

### Conclusion

This paper provides a conceptional framework and a comprehensive understanding for complexity in palliative care. On the level of the individual care situation, the systemic view can help to understand and shape situations and dynamics. On a higher hierarchical level, it can support an understanding and a framework for the development of care structures and concepts.

The framework and the identified system elements can be used as a basis for the development of a classification of complexity in palliative care, drawing on a differentiation of patients according to their care needs. Relevant outcome measures mirroring the identified system elements have to be identified and implemented in clinical practice. The consideration of phases of illness as an attractor may constitute a promising starting point for the operationalisation of complexity in research, clinical practice, and health policy planning. Further elaboration of relevant parameters and suitable methodology to adequately model complexity should be pursued in future research and theory-based deliberation among interdisciplinary experts.

## Additional files

Additional file 1: COREQ Reporting Checklist. (DOCX 18 kb)

Additional file 2: English translation of topic guides. (PDF 142 kb)

Additional file 3: List with illustrating citations for each system element – translated original data. (DOCX 47 kb)

## Abbreviations

AN-SNAP: Australian National Subacute and Non-acute Patient Classification
CAS: Complex Adaptive System
DRG: Diagnosis Related Groups
IPOS: Integrated Patient Outcome Scale
PC: Palliative Care
WHO: World Health Organisation
