# Supplementary material for: Understanding complexity – the palliative care situation as a complex adaptive system
Source: BMC Health Serv Res. 2019 Mar 12;19:157. doi: 10.1186/s12913-019-3961-0 (PMC6417077; doi:10.1186/s12913-019-3961-0)
Supplement: Supplementary file 1 — COREQ Reporting Checklist. (DOCX 18 kb) [file 12913_2019_3961_MOESM1_ESM.docx]

**COREQ Reporting Checklist**

| Domain 1: Research team and reflexivity | |
| --- | --- |
| Personal Characteristics | |
| 1. Interviewer/facilitator | Farina Hodiamont (FH), detailed in methods section “Data collection and analysis” |
| 1. Credentials | Magister Artium (M.A.) equivalent to a Master degree |
| 1. Occupation | Research Associate |
| 1. Gender | Female |
| 1. Experience and training | Not reported due to space limitations: University degree in Sociology, conception and realization of several qualitative interview studies. |
|  |  |
| Relationship with participants | |
| 1. Relationship established | Not reported due to space limitations: Between some participants and research team members a relationship was already established prior the project start. The relationships were of a neutral to friendly professional character. |
| 1. Participant knowledge of the interviewer | Not reported due to space limitations: In four cases the interviewer was known to the participant prior to the study. These relationships derived from a prior working position and a prior research project. |
| 1. Interviewer Characteristics | Not reported due to space limitations: The interviewer has a research interest in complexity and costs in palliative care, especially in the question what makes situations of patients in palliative care complex. |
| Domain 2: Study design | |
| Theoretical Framework | |
| 1. Methodological orientation and theory | Stated in the methods sections “study design”, “sample and data collection”, and “data management and analysis”. Data were analysed using the Framework approach. |
| Participant selection | |
| 1. Sampling | Stated in the methods section, “sample and data collection”, purposive; by suggestions of the research team, collaborating partners and representatives of the German Association for Palliative Medicine. |
| 1. Method of approach | Not reported due to space limitations: An invitation letter to take part in the study was sent by email by the project lead (Prof. Bausewein). In three cases a personal pre-contact on an international congress was used to announce the study and the impending interview- invitation. In case of acceptance a date for a face-to-face interview was made either by email or telephone. |
| 1. Sample size | Reported in the first paragraph of the results section and table 1. |
| 1. Non-participation | Reported in the first paragraph of the results section. |
| Setting | |
| 1. Setting of data collection | Reported in the last paragraph of the methods section “sample and data collection”. |
| 1. Presence of non-participants | Not reported due to space limitations: No one else was present besides the participants and the interviewer. |
| 1. Description of Sample | Reported in the first paragraph of the results section and table 1. |
| Data Collection | |
| 1. Interview Guideline | The development of the interview guide is described in the methods section, “interview guide”.  The interview guide (in German language) is available from the authors on request. |
| 1. Repeat interviews | Not reported due to space limitations: No repeat interview was necessary. Interviews were recorded and conducted by a trained researcher. Participating experts were asked to be contacted again in case of any uncertainties or lacks of information becoming evident subsequent to the interview. The project team did not have to make use of this possibility. Also no technical problems occurred which could have prompted the necessity of a repeat interview. |
| 1. Audio/Visual recording | Reported in the methods section, “data management and analysis”: All interviews audio recorded and transcribed for further analysis. |
| 1. Field notes | Not reported due to space limitations: A field-note form was filled in by the researcher after each interview, covering the following topics: relationship between researcher and interviewee, interview setting, account on interruptions, interview atmosphere, perceived moods or emotions of interviewee, difficulties in carrying out the interview, comments on content, feelings of the researcher. In case of a distinctive behaviour or strong emphasizing on certain topics, respective memos were added to the transcript. |
| 1. Duration | Reported in the first paragraph of the results section: Interview duration ranged between 19 and 113 minutes with a mean duration of 58 minutes. |
| 1. Data saturation | The proceeding to achieve saturation is reported in the methods section “sample and data collection” |
| 1. Transcripts returned | Not reported due to space limitations: Transcripts were not returned to participants. Statements and descriptions were however, continuously confirmed by the interviewing researcher during the interview in order to guarantee the correct understanding. |
| Domain 3: Analysis and findings | |
| Data analysis | |
| 1. Number of data coders | Reported in the methods section, “data management and analysis”. |
| 1. Description of the coding tree | Only by representing the findings, due to space limitations. Available from the authors on request. |
| 1. Derivation of themes | Reported in the methods section, “data management and analysis”. |
| 1. Software | Reported in the methods section, “data collection and analysis”: NVivo 10. |
| 1. Participant checking | Not reported due to space limitations: Participants were not asked to provide feedback on the findings. |
| Reporting | |
| 1. Quotations presented | Due to space limitations no citations are be used for illustration within the paper. A list with illustrating citations for each system element is provided in the online appendix. |
| 1. Data and findings consistent | Yes |
| 1. Clarity of major themes | Yes, we present the major themes. |
| 1. Clarity of minor themes | Yes, as far as space limitations permit, we discuss minor themes, too. Table 2 shows all minor themes and their relations. |
