# Supplementary material for: Understanding complexity – the palliative care situation as a complex adaptive system
Source: BMC Health Serv Res. 2019 Mar 12;19:157. doi: 10.1186/s12913-019-3961-0 (PMC6417077; doi:10.1186/s12913-019-3961-0)
Supplement: Supplementary file 2 — English translation of topic guides. (PDF 142 kb) [file 12913_2019_3961_MOESM2_ESM.pdf]

Cost and Complexity in Palliative Care in Germany
Interview guide for clinical experts

Background information on care institution

- Which are the predominant conditions of patients treated here?
    - *Only patients with malignant diseases? Which non-malignant conditions?*
    - *Do you provide chemotherapy, ventilation, or radiotherapy?*
- What is the proportion of patients being discharged every year?

Complexity

- Based on your experience – how would you describe a complex patient situation in your setting / your institution?
- Here are two case vignettes (presentation of the vignettes). If you look at these cases, what do you think about the complexity of these patient situations?
- What do you think are the main factors in these two cases that determine the degree of complexity of each situation? *(note factors on flashcards)*
- Which other factors do you consider potentially affecting the level of complexity of a patient's situation? *(note factors on flashcards)*
    - *Special symptoms or needs?*
    - *In addition to symptoms/needs, which other individual patient and/or family characteristics might influence the level of complexity?*
    - *If not mentioned by the interview partner: in which direction (higher or lower level of complexity) do factors influence complexity?*
- Do the factors you mentioned apply to all patients?
- Are certain factors more important than others for determining the degree of complexity? If yes, which ones?

Resources

I'd like to talk to you about resources now. The concept of resources does not refer to the patients' ability to cope with life and the illness, but to the resources that need to be provided by the care institution and the health system.

- In the international discussion it is assumed that a patient with a higher degree of complexity needs more resources than a patient with a lower degree of complexity.

Do you think that this assumption also applies to Germany? Thus, do more complex patients require more resources?

a) If *„no“*

- Why?
- If I understand you correctly, the factors you named earlier to describe complexity are not the factors that increase resource needs. Can you tell me which factors increase resource needs? (-> add and change flashcards)

b) If „yes“

- Could you give examples?
- To what extent do the factors that you indicated to determine complexity influence the level of resource needs, respectively, can the same factors be regarded as increasing resource needs? (refer to flashcards)
- Which of these factors would you not consider to increase resource needs and why?
- Can you think of any other factors increasing resource needs?

- The following factors have been identified as the main drivers of resource use in palliative care (show cards with Australian factors): problem severity, functional status, age, phase of illness.
  Do you think that these factors may be main drivers for resource needs in Germany, too?

  a) If „No“ – factor is not considered to describe resource needs: Why not?
  b).If „Yes“ – factor is considered to describe resource needs: Please explain.

- Which additional/other factors should be taken into account?
  If not mentioned: What do you think about the lack of diagnosis in the Australian factors?

Resources – Reimbursement

So far we talked about complexity and resource needs. Now I would like to talk with you about reimbursement.

- How are services reimbursed in your institution, or which reimbursement system is used here?
- What strengths and weaknesses do you see in this reimbursement system?
- Do you see any possibility for misplaced incentives with this system? If so, which are these?
- How appropriate do you think this reimbursement system is?
- From your point of view, how cost effective is this reimbursement system and why?

- Do you think that reimbursement should be based on individual use of benefits (as in the DRG system) or would you prefer a structural funding (as in the case in per-diem rates)? And why?

Outcome Quality

We think that some of the factors describing complexity of a patient situation may also describe outcomes or outcome quality, if collected at a second point in time.
Explanation: Measured at one point in time, a specific need describes the complexity of a patient situation. If the same need is measured again at a later time point, it can be judged whether things have improved. This would then lead to a statement about outcome quality. A pain score, for example, gives information about the need for pain management at the time of the first measurement. At a second point in time, the pain score provides information whether the therapy was successful.

- Do you think the distribution of resources should be determined by needs or rather by outcome quality? And why do you think so?

Comments

- Is there anything else you would like to add? Any comments?

Cost and Complexity in Palliative Care in Germany
Interview guide for experts with a health policy and financial background

Experience

- How are you involved in the reimbursement system for palliative care in your work / What do you know about it?
- What experience do you have with reimbursement of palliative care services by DRGs and by per-diem rates?

Opinion on Reimbursement System

- What strengths and weaknesses do you see in the current reimbursement system for palliative care?
- How appropriate do you think is the current reimbursement system for palliative care in Germany?
- Do you consider this reimbursement system to be cost effective and why?
- Do you think that reimbursements should be based on individual use of benefits (as in the DRG system) or should structural funding be available (as in the case in per-diem rates)? And why?
- If you compare reimbursement models in palliative care with other areas of the health care system, do you see any particularities or specific challenges, and if so, which ones?

Complexity

In case of the DRG system, the reimbursement is based on diagnoses and procedures (OPS). Considering the aim of treatment and care in palliative care, it is discussed whether diagnosis and OPS are actually suitable in this domain.
In other countries, such as United Kingdom and Australia, where palliative has long been established, an alternative system focusses on the complexity of a patient situation and not on diagnosis.

- What would you understand by the complexity of the patient situation?
- Here are two case vignettes *(presentation of the vignettes).* If you look at these two cases, what do you think about the complexity of these patient situations?
- What do you think are the main factors in these two cases that determine the degree of complexity of each situation? *(note factors on flashcards)*
- Which other factors do you consider potentially affecting the level of complexity of a patient's situation? *(note factors on flashcards)*

Resources

These factors have been identified as the main drivers of resource use in palliative care *(how cards with Australian factors): problem severity, functional status, age, phase of illness.*

- *Do you also consider these factors as the main drivers for resource needs in Germany?*
  a) If „No" – factor is not considered to describe resource needs: Why not?
  b).If „Yes" – factor is considered to describe resource needs: Please explain.

- *Which additional/other factors should be considered?*
  If not mentioned: What do you think about the lack of diagnosis in the Australian factors?

Documentation of Service Delivery

- We would like to develop a methodology to document the service delivery in palliative care and to calculate real costs per patient. Which aspects should be considered in such a method of collecting data on service provision and cost calculation?
- To what extent are aspects of the InEK methodology currently applied to record service provision in intensive care and surgery, transferable to the recording of service provision in the palliative care unit?
- What should be changed or complemented?

Inter-Setting-Comparison

- Do you see any relevant differences in resource use and costs between palliative care units and inpatient hospices?

Outcome Quality

We think that some factors describing complexity of a patient situation may also describe outcomes or outcome quality, if collected at a second point in time.
Explanation: Measured at one point in time, a specific need describes the complexity of a patient situation. If the same need is measured again at a later time point, it can be judged whether things have improved. This would then lead to a statement about outcome quality. A pain score, for example, gives us information about the need for pain management at the time of the first measurement. At a second point in time, the pain score provides information whether the therapy was successful.

- Do you think the distribution of resources should be determined by need or rather by outcome quality? And why do you think so?

- How could the measurement of outcome quality be integrated into a model of resource allocation?

Comments

- Is there anything else you would like to add? Any comments?
