# Supplementary material for: Understanding complexity – the palliative care situation as a complex adaptive system
Source: BMC Health Serv Res. 2019 Mar 12;19:157. doi: 10.1186/s12913-019-3961-0 (PMC6417077; doi:10.1186/s12913-019-3961-0)
Supplement: Supplementary file 3 — List with illustrating citations for each system element – translated original data. (DOCX 47 kb) [file 12913_2019_3961_MOESM3_ESM.docx]

**Additional file 3: Illustrating citations for each system element of the complex adaptive system of a palliative care situation and the environmental factors of the system environment.**

| **SYSTEM PATIENT** | |  |  |
| --- | --- | --- | --- |
| **Physical Subsystem** | |  |  |
| **Physical symptoms and clinical signs** |  |  |  |
| Physical symptoms in general | *“Well, a symptom, even a single symptom can affect a patient so severely and on many levels that it may result in a complex care situation. Maybe only temporary, and when the symptom is treated, the situation improves again.” (I-28 – nurse, palliative care unit)* |  |  |
| Symptoms resulting in emergency situations | *“All those symptoms which most likely necessitate crisis interventions, I think, those result in a complex or at least a potentially complex care situation. Because those crises not only affect the patient but also his social environment, his family, his children. Well, because of a crisis the overall situation may change rapidly, may even change towards dying – in terms of that a crisis may cause dying.” (I-28 – nurse, palliative care unit)* |  |  |
| Physical impairment | *“Sure, in such situations it is always expected that someone needs more and more help. And this is very difficult especially for quite independent people.” (I-13 – nurse, palliative care unit)* |  |  |
| Pain | *“Well, it is like it is, I can’t say I’m doing pain management and give drug XY and the pain is relieved and that’s it. This is connected to so many other things as it is for example portrayed in the concept of total pain. I can’t express it better.” (I-34, physician, palliative care service/palliative care community team)* |  |  |
| Breathlessness | *“Breathlessness, if not managed, is the symptom that probably affects carers and the team most and makes them suffer.” (I-4 – physician, palliative care community team)*  *“This symptom always creates problems, when for instance the patient is released from inpatient to outpatient care. Often, patients feel save in the inpatient care setting, where someone is present at any time, when the breathlessness gets worse and so on. And they can hardly imagine that this is possible at home. Even though you make them all sorts of offers. So, the idea alone to be at home in a situation of breathlessness makes it impossible for them to be discharged. Or it makes it extremely difficult.“ (I-18 – nurse, palliative care unit)* |  |  |
| Complex wounds | *“(..) because sometimes we have patients with half or completely open and incrusted ventral or dorsal thorax. On the one hand this makes this carapacious feeling for the patients. But what is more important is that we manage a good wound care, that our wound managers manage a good wound care, so it doesn’t smell, it doesn’t constantly leak out so that patients are able to receive visitors. In case there are children for example or other family, that they [authors remark: the patients] are able to restore their outer appearance the way they want it. Well, there are families, I have known some, who see this, and can attend the wound as if it was a normal small wound and this person looks to them like he or she used to. But that’s very rare. (I-4, physician, palliative care community team)* |  |  |
| Personality change | *“Confusion up to psychotic symptoms, hallucinations, feeling threatened, these are situations which are in an outpatient situation often nearly unbearable for relatives (12 -* general practitioner*, palliative care community team/ generalist palliative care in the community)* |  |  |
| Cognitive impairment | *“Well, if this disorientation, this shifting, this being confused, if this is combined with an unstable social situation, it increases complexity.” (I -1 – physician, palliative care unit)* |  |  |
| Limited ability to speak and hear | *“And what I find particularly distressing, are deaf people, people with profound hearing loss. Because we communicate using voice, using speech. And if somebody doesn’t know you and is weak, he can’t read your lips. And often they can’t read anymore, either. And then the wildest ideas arise, what is going to be done to them, guinea pig or something like that.” (I-10* general practitioner*, hospice/generalist palliative care in the community)* |  |  |
| **Primary diseases and disabilities** | |  |  |
| Cancer | *“Or the other way around, if someone has cancer, everyone suspects right away that this is life limiting. At the back of your mind are those things, things which will follow. Fear, pain, these are the associations.” (I-12 nurse, palliative care unit)* |  |  |
| Non-oncological disease | *“And chronical diseases are rather related with loss of quality of live and I’m not able to do this anymore, and I’m not able to do that anymore, and so on. But apart from this, everything is fine, and it is not associated with dying. And that’s the problem I see. This is why coping with the disease can be seen as the main indicator, particularly in terms of chronic organ failure?” (I-14 – nurse, palliative care community team)*  *“Sometimes I have the impression that patients with a non-oncological diagnosis are more complex than those with an oncological diagnosis because we are possibly better trained and experienced to assess the course of the disease. Among our non-oncological patients the disease trajectories are very various. Often they take drugs we don’t know very well. And often we have to familiarize ourselves with the disease. This often results in complex interrelations.” (I-27 – physician palliative care unit/palliative care service)*  *“But this also refers to another level and to what is there on the level of the health care system. This is also a point – many things don’t exist for these patients. There are palliative care community teams but it is a real challenge for a COPD patient to get admitted and not get sacked again after a week.” (I-29- physician, palliative care unit/palliative care service)* |  |  |
| Short prognosis | *“And what also came to my mind, well, we currently have a young patient and he suffers from such an aggressive, rapidly progressing disease. I currently have three or four patients with a diagnosis from 01/2015. They didn’t have any chance at all to get used to anything.” (I-20 – physician, palliative care unit)* |  |  |
| Long-term and uncertain prognosis | *“This is not really the case with patients with cardiovascular diseases because they have a much longer course of disease also regarding the point in time where physicians agree to stop treatment. And you always have – and this also makes the prognosis with patients with cardiovascular disease quite difficult – you never know is this the point where an intervention isn’t helpful anymore is this not the point, yet?” (I-13 – nurse, palliative care unit)* |  |  |
| Disabilities | *“Another case is patients with multiple disabilities or severs disabilities. We had the problem several times now, that people who already get personal assistance at home, that the funding body refuses to pay that for the hospital setting because the patient’s care is provided there. However, their care is often so time-consuming that this is only realisable with 24-hour care availability. (I-27 – physician, palliative care unit/palliative service)* |  |  |
| ***Therapeutic measures*** | |  |  |
| Medication | *“Or where interactions between drugs and diseases are possible, which are not predictable, not at first, this would be a complex matter.” (I-37 – Heath care system researcher)* |  |  |
| Technical Aids | *“For example a tracheostoma. This can per se create a lot of problems, definitely at home but also in nursing homes. Well, the more technology, with ventilator etc., the more difficult it becomes at home to manage all that. You need people who can master this; Elsewise, there will always be problems.” (I-18 – general practitioner; generalist palliative care in the community)* |  |  |
| Ethical challenging decisions | *“And possibly, and this would be such a typical patient, if it gets worse, it would be possible to think about a palliative sedation. Would be a new aspect of complexity, I think, because this isn’t done just like that. Well, we don’t decide – ok, now we induce a palliative sedation and then everything will be fine – this is an aspect which must be discussed with the patient, with the relatives and the different professionals. This isn’t done overnight. (I-3 – nurse/hospice management, hospice)* |  |  |
| ***Psycho-spiritual Subsystem*** | |  |  |
| Psychological factors in general | *“Or if I take a look on the people currently dying, there is a number of refugees. Especially in [name of a German city – alteration by author] has lots of refugees. And they have memories in their head, of war or of escape and all these experiences. Even if nothing happened to themselves, they heard and saw a lot of things. Those often come to mind in the end of life and require much more or a different support than people who grew up in peaceful and problem-free situations. (I-6 – palliative care unit)* |  |  |
| Anxiety | *“Another aspect is the patient’s psychological situation. So, if he doesn’t even have that severe medical problems but is very afraid and uncertain, than this is the reason for complexity. Because it is really hard to support the person in the home care setting and you need to try to transfer him in a more protected care setting, right. In a hospice or an inpatient facility.” (I-5 – social worker/coordinator, palliative care community team)* |  |  |
| Desire for hastened death | *“The desire to die, well massive desire to die, if someone says I don’t want any of this anymore, give me something. And to understand what does that mean. Is it an actual desire to be dead or is it a wish not to live anymore in the given conditions?” (I-38 – expert in health policy, all settings)*  *“Well, if within the discussion on assisted suicide the focus of care would for instance mean to expect the care team to euthanize the patient or assist suicide. Than this means complexity for the team to cope with that.” (I-12 –* general practitioner*, palliative care community team /generalist palliative care in the community)* |  |  |
| Understanding and comprehension of the disease and situation | *“For one, there is the ethical question; the patient refuses the dialysis, there you have to consider whether he is sufficiently informed about what exactly this means and how this situation can be attended.” (I-38 - expert in health policy, all settings)* |  |  |
| Depression | *“Well, if there is already, depending on how severe or pronounced the depression or the depressive phases are, which she develops, or whether it is an episode or a continuous problem, however, than it is already complex from the start. If the woman cannot be active due to her illness, not because of the bone metastasis and the breast cancer but because of the depression, then she needs support beforehand and that makes it difficult. Because all this coping with the disease and life management probably goes or is already out of joint.” (I-14 – nurse, palliative care community team)* |  |  |
| Mental comorbidity | *“Well, addiction problems, we of course have people with addictions, that shouldn’t be forgotten. I am not only thinking of heroin addicts but also of alcohol, benzodiazepine- and psychotropic drugs. That is certainly an issue, one we currently have on our ward again. (…) Because this makes it difficult to make and stick to agreements, in terms of we agree to it is that and that much drugs per day. But also what they take secretly or additionally without us knowing. And those are situations when we ask how can we work together, if we cannot rely on certain things. What additional drugs do they take, right? If you have that impression, well, and it is also getting difficult because people get aggressive, if they don’t get their benzodiazepine, what we don’t even know sometimes. So, that is making it more difficult, addictions.” (I-23 – Physician, palliative care unit/palliative care service)* |  |  |
| Patient’s spiritual situation | *“But does the foundation I built my life on, does that carry me unto death? If that is given, whatever it may be, then many things get easier. If not, then it becomes complex.” (I-42 – physician, palliative* care unit*)*  *“Recently we had an extremely complex situation. That was a woman with a certain world view, which was anthroposophical and she refused all treatments.” (I-8 – nurse/hospice management, hospice)* |  |  |
| **Internalized rules of the patient** | |  |  |
| Personality | *“Or a patient who has a problem with allowing people into his apartment, this makes it very complex as well, because, well, this willingness just needs to be there, in order to make that work.” (I-2 – nurse, palliative care community team)* |  |  |
| Prior experiences with health care system | *“And of course, if communication went wrong before. Well, if they come to us with incorrect information, misinterpretations, I’d say. This white coat syndrome, when they have the opinion that physicians are always wrong and nothing works with them. So, if they have already made negative experiences, that makes a palliative care situation much more complicated. (I-23 – physician, palliative care unit/palliative care service)* |  |  |
| **Self-organisation of the psychosocial subsystem** | |  |  |
| Coping with disease and situation | *“Well, I don’t know. The day that Mr Braun has been told: You have renal failure and we would recommend you dialysis. At this point, complexity was most likely more severe. But some things have been sorted out since, what he assessed for him personally. (I-34 – physician, palliative care service/palliative care community team)*  *“And to prepare this, that they are informed and he, this Advance Care Planning, where would he like to stay, how much is his wife able to endure, and this was very difficult because both of them were always blocking out. They built a wall around themselves, it was extremely difficult. And at the same time we had to prepare the bleeding. And we made it but that was really last minute and we could have made it earlier.” (I-4 – physician, palliative care community team)* |  |  |
| **Socio-cultural subsystem** | |  |  |
| Different cultural background | *“I have the feeling that distress is defined and perceived differently and that also the relationships with and within the family are different than in situations we are familiar with. Well, that they don`t talk as much. Sometimes we can dissolve this by offering them, and also by telling them that there are cultural differences. Here we deal with things like this and they deal with things like that. And how shall we do it now that they are here? The way we do it or the way they do at their home. And if it is possible to do it the way we are used to than it is easier. Otherwise we’ll have difficulties with our team members since they are used to speak openly so that it’s hard for them if that isn’t possible.” (I-6 – Physician, palliative care unit)* |  |  |
| Language barriers | *“There are not that many here, but sometimes we have German-Russian families here. (…) And that can cause language problems and then it’s difficult with the translation because generally someone from the family translates. And it is difficult. It only happens rarely that we get an external translator. In some situations it makes sense but especially if is about things which can’t wait than it’s not possible at all. Then you simply have to react individually and involve people who are on site. And we don’t always know what is being translated. And they grew up here, and they understand what we want but sometimes they have the feeling they cannot say it the way we do. And this we can’t control.” (I-6 – physician, palliative care unit)* |  |  |
| Educational level/intellect | *“Or at the moment we have a family, they are illiterates. Well, and then you have to think about how you can actually provide them with the information what to do in which situation.” (I-5 – Social worker/coordinator, palliative care community team)* |  |  |
| Financial situation | *“If , well let’s say, existential matters are on stake like for instance the dissolution of once home, eviction, because someone couldn’t pay his rent.” (I-2 – nurse, palliative care community team)* |  |  |
| **Characteristics of the system patient as an agent** | |  |  |
| Age | *“And when there is an old person who is palliative and who sort of already dismissed everyone around him into independence; and who is the dependent one himself now, well, he doesn’t necessarily need to be replaced.” (I-31 – nurse, palliative care service)*  *“Young patients always, always make it difficult for the treating team. There is a pretty good study, in which it was analysed how physicians estimated the life expectancy of severely ill and dying people. And it was very clearly shown that with people of the same age, or people with whom the professional has much in common, life expectancy was expected to be on average one month higher than in people with whom you don’t have much in common. Yes, those are things making it complex.” (I-38 – expert in health policy, all settings).* |  |  |
| **SOCIAL SYSTEM** | |  |  |
| **Characteristics of carers** | |  |  |
| Carer’s health | *“And someone says; I don’t even know how I can manage to get my husband onto the commode. (…) Well, if the carer himself isn’t at good health, be it an addiction or could also be another chronic illness limiting the carer.” (I-12 –* general practitioner*, palliative care community team/general palliative care in the community)* |  |  |
| Carer burden | *“But also this very healthy and fit woman can have a breakdown and decompensation, and can then herself react in a depressive way. And then this pillar of support falls away as well.” (I-41 – expert in health policy, palliative care unit)*  *“Or if we keep in mind that 50 percent of the overall working hours on a palliative care ward are spent on relatives. For me, it should be an important criteria because we know that not only it stabilizes the patient and helps him but also it is an enormous preventive measure for relatives who would otherwise be sent into rehab after nursing someone for a long time.” (I-38 – expert in health policy, all settings)* |  |  |
| Level of information and understanding | *“And regarding the relatives also a lack of information, they are not really informed about certain situations because often this is very time-consuming. Yes, what does it mean when an old person stops eating and drinking, yes. Or there are imaginations that someone starves to death or dies of thirst. Than, if someone doesn’t drink anymore, than terms like kidney failure are being dropped but nobody explains what this actually means. And people tend to have this sort of mental cinema, and the film director always makes horror movies, because it is not explained what kidney failure means, what it means to die of pneumonia.” (I-12 – nurse, palliative care community team/generalist palliative care in the community)* |  |  |
| Family's coping with patient's disease | *“And then the wife in all her coping with the disease, managed to convince the patient to start a palliative chemo therapy, which was offered to him without much consideration. (…) And at some point the patient said: My wife is closest to me so I am adding her to my last will pool, she should be happy as well, she shouldn’t blame herself to have missed out on something, so I am just doing the chemo, now.” (I-31 – Nurse, palliative care service)*  *“And it also varies regarding the social environment. On the one hand they were always capable to support that, but than they often have a problem with things ending in terms of that they don’t want to admit that it is not again the get up and go person, that’s how these people often are, well that this is not happening again.” (I-22 – nurse/coordinator, palliative care community team/generalist palliative care in the community)* |  |  |
| **Social roles and functions of individuals involved** | |  |  |
| Relatives cared for by patient | *“And every now and then we have patients, where for instance their relatives have dementia. And then the one, who actually always realized the care at home, gets sick all of a sudden and lives together with his dement wife. And than you have to think about how you can support this care system so that both can handle it. Or how could you transfer them into a more protected living situation, where both of them can stay until they die. Those are things where it is not about the medical side but about organisational aspect and supporting the social system.” (I-6 – social worker/coordinator, palliative care community team)* |  |  |
| Minor children | *“Well, from my point of view it’s really small children. For me, that makes it complex at once, because there is always the question of who is taking care of the children, who can take care of the children after the patient’s death? Will there be any family structure at all? I think minor children make the situation difficult at once.” (I-19 – physician, palliative care service)*  *“Well, I can imagine, or can tell from experience, that pubescent children can escalate a situation for the patient and by that of course increase symptom burden.” (I-14 – Nurse, palliative pare community Team)* |  |  |
| Difficult underlying family situation | *“And families who had problems before. Whether it is a couple, who was actually already in the process of getting divorced and now one of them got sick. Be it a family who is terribly fighting. Well, once there were four sisters and their mother was the patient, and the sisters were fighting so hard I really thought I’d had to protect my office so they won’t disassemble it. And then a lot of conversations were necessary to find out what the sisters wanted, what the patient wanted or what would be good for her. And then one of the sisters took over the care and the others didn’t trust her, she wouldn’t be able to manage and would do everything wrong anyway. And this was all very difficult.” (I-19 – physician, palliative care service)*  *“Well, if there are for instance unsolved and unspoken issues in families. So, if patient and carers carry out conflicts here, then it is not uncommon that someone takes sides for either the patient or the family. And often these conflicts can lead to conflicts within the team. This makes a treatment, a support complex.” (I-27 – physician, palliative care service)* |  |  |
| Differences of opinion between patient and family | *“Well, so this son from Hamburg might come and say, what we are doing here is all wrong and we will do it completely different. We will go and see Professor X and he’ll do great. And then it also becomes difficult, if the actual patient says, I don’t want that. So this is an important factor for complexity.” (I-18 –* general practitioner*, generalist palliative care in the community)*  *“Well, differing information levels are a very important and complex problem when a patient knows more than the relatives do and vice versa. And this makes it very complex, I think.” (I-23 – Physician, palliative care unit/palliative care service)* |  |  |
| Difficult communication between team and family | *“This was one reason why we transferred him to a hospital with this complication. To have another official voice, well, to have two official statements that he is indeed, that we independently of one another think that he is in the terminal phase. So, actually because of legal reasons because I knew that would be reopened.” (I-4 – Physician, palliative care community team)* |  |  |
| **SYSTEM TEAM** | |  |  |
| **Structural characteristics of team** | |  |  |
| Number of professions | *“Well, I have a part time social worker and when she is on vacation I call the social services belonging to the clinic. And this are nurses who have this additional qualifications, which you need for discharge management. And even here you can feel the big difference, yes? To have a constant person, who belongs to the team who is present and involves herself into the discussion processes. And otherwise, you have one person one day and another one the next. This makes it very difficult.” (I-20 – physician ,palliative care unit)* |  |  |
| Number of team members | *“And now he gets paranoid and aggressive conditions. Such things are difficult. What I would wish for in such a situation, and what would be very helpful to us, would be more staff available. If I could tell somebody to sit with him, who would just be there and distract him somehow and positions him a little or so.” (I-10 – General practitioner, hospice/generalist palliative care in the community)* |  |  |
| **Characteristics of team-members as social agents** | |  |  |
| Team burden | *“But, this also refers to the team. What I am missing is the complexity within the team which is maybe caused by a patient. So, how the team is burdened? And this increases complexity even though the patient’ complexity remains the same.” (I-4 – physician, palliative care community team)*  *“And that makes ALS-patients so difficult. Most teams only endure one ALS-patient, because they often convey the impression you didn’t do enough for them. You leave them behind helplessly. These patients are very burdensome for a team in the sense that they cause a permanent guilty conscience.” (I-10 – general practitioner, hospice/generalist palliative care in the community)*  *“Well, this is a 42-year-old patient with a 3-year old daughter, right? And we notice that team members who are in a situation like this themselves, always say: Um, that could be me.” (I-8 – nurse/hospice management, hospice)* |  |  |
| Qualification within team | *“What probably adds to that is, let’s say, the difficulty, lack of knowledge, lack of experience on our part, on part of the professionals, the care takers, the care system, regarding non-cancer patients.” (I-29 – physician, palliative care unit/palliative care service)* |  |  |
| **Relations producing complexity** | |  |  |
| Cooperation with/ coordination of external service providers | *“Well, when there is great need for coordination, not only concerning the care directly on the patient, but also the coordination concerning the care system. So, how do I bring together the individual service providers and responsible persons so they collaborate well. And I need to rely on this when I care for a patient. Well, there is the individual case level which can be complex and there is the system level which is generally complex. And bringing this together without it being an additional burden for the patient, this is an essential task area of coordination, for example.” (I-28 – Nurse, palliative* care unit*).*  *“What is probably still missing is, when we are not openly welcomed by other services. When, for instance, a GP has the impression we take away his patient; or a nursing care service says, the carer from a palliative care community team doesn’t have a say; Or a nursing home says, we can manage on our own. And this would be, well, this competition.” (I-2 – nurse,* palliative care community team) |  |  |
| Knowledge, skills and attitude of external service providers | *“And of course what makes it worse is the ignorance about what palliative care actually is. So, the lack of information on palliative care, which is transferred from team to the patient. Well, palliative care, dying, right? So, when they come and are like: Oh, is it time already? Yes? This is how they partially are informed, falsely informed. These is this false information.“ (I-23 – physician, palliative care unit/palliative care service)*  *“A particular requirement, especially in palliative care the pain management, is not yet developed sufficiently in acute care hospitals. There are special requirements. Any other physician hesitates to give 100 milligrams of Morphine because he thinks “I’ll kill the patient, I’ll make him addicted”. And well, in an acute care hospital, the general physician, the general environment, also the nurses, they would not be able to meet the requirements in such a particular situation” (I-33 – health care system researcher)* |  |  |
| Divergent assessments of situations by team and patients and carers | *“On the other hand, something which seems little to us turns out to be a lot for the family because for them it is difficult, severe and unbearable and we might not understand why it this way with this family. And the same situation with another family is completely unproblematic.” (I-6 – physician, palliative care unit)* |  |  |
| **ENVIRONMENTAL FACTORS** | |  |  |
| **Factors of space and time** | |  |  |
| Care setting | *“Those are factors of complexity, and there I would like to make a point, especially coming from the home care sector, that it shouldn’t be reduced to the medical component but also consider the related psychosocial component which can contribute significantly to increase complexity.” (I-29 – expert in health care policy, palliative care community team)/generalist palliative care in the community)*  *“(…) in my opinion it is very important that the patient is well-cared outside, at home. Is he in good hands within the family? Is there someone to take care of him? Are there any relations, yes, no? Because often we have the problem that the patient could be released. From the medical side there is not much else to do anymore but we know for sure that there is nobody.” (I-24 - expert in financing, palliative care unit /palliative care service)* | |  |
| Patient's living situation | *“Well, someone lives in an area which is hard to reach, where there is nothing around and where the care service can’t drive by like that. And that makes the whole situation more difficult. Things have to be planned in advance then. Situations have to be better thought through and planned in the long term. You have to think what to do if something does not work out as expected. Who will go? How long is that allowed to take? That is this advance care planning thing, that would be such a situation.” (I-6 – physician, palliative care unit)* | |  |
| Nighttime | *“The situation would most likely exacerbate at night because it can be assumed that all these symptoms and aspects of the patient situation intensify at night.” (I-3 – nurse/hospice management, hospice)* | |  |
| Local care provision | *“(…) to create a palliative care structure means I need an entire palliative care culture in the hospital. Not only in one hospital unit. This is why it isn’t enough to have a palliative care unit. Because that way I only reach a fraction of the patients who are in need of palliative care.” (I-41 – expert in health policy, palliative care unit)* | |  |
| **Structural characteristics of internal and external cooperation** | | | |
| Time of integration of palliative care (PC) | *“And what is clearly also a reason for complexity is that the palliative care team often is informed far too late. That means that in a very short amount of time, well, we have a very short timeframe. (…) Yes, too late within the course of the disease or too late in regards to symptom control, too late for us as a task force. In other words, we come and within one hour or one day we have to save everything. And this costs us a lot of energy. We come and the whole mess comes at us right away. And then we often don’t have the time to reflect in peace about the situation.” (I-23 – physician, palliative care unit/palliative care service)* | |  |
| Admission criteria for care | *“If relatives are completely overwhelmed, and yet there is no bed on the palliative care ward or in the hospice available for the patient, because the patient either doesn’t fulfil the admission criteria, or because there is no free bed. And if suddenly and spontaneously the care system at home collapses, maybe also because things were actually known but no one could yet name and address the issue in order to change things.” (I-2 – nurse, palliative care community team)* | |  |
| Structural characteristics of the organisation | *“I’m sure when I say that most of the time we are able to manage the psychological problems, and health problems, the physical symptoms, that there are of course teams who have much more difficult patients because they affiliated to a University Hospital.” (I-2 – nurse, palliative care community team)* | |  |
| **Structures of the German Health Care System** | | | |
| National palliative care (PC) system | *“And then there is also the complexity in terms of care provision. Well, our health care system is very complex per se, let alone because of the different care providers, the different regulations, contracts, laws, civil codes and jurisdictions. And this type of complexity can also affect the patient’s situation. Well, when it is not clear who is responsible for what.” (I-28 – nurse, palliative care unit)*  *“Complexity also results from the patients’ social situation, when they are changing back and forth between inpatient and the home care setting, who remains responsible for what? That’s quite a jungle for patients and relatives. (I-34 – physician, palliative care service/palliative care community team)* | |  |
| Funding of palliative care (PC) in Germany | *“Well, there are, if they are coming to the palliative care ward or to the hospice and dosages are being adjusted, or drugs are being completely withdrawn, and then they suddenly recover. And if people are in a hospice, these are other circumstances, than you cannot keep them because only three months are getting refunded, I think. And than they actually have to go back home but the family, the home is not prepared for that.*  *Well, how to manage a situation where the patient recovers but is still dying, but exceeds the time and cannot stay in the hospice. This is when the patient’s suffering really starts, because this becomes a ping-pong action then. And then there is something, like a complication, stomach pains or at home they are overwhelmed, the care service as well, hence – hospital.*  *For the patient this is a undignified situation, I think. This is why we need regulations how to manage those situations. We don’t have that in Germany. (I-33 – Health care system researcher)* | |  |
